# A Note on Electrical Treatment

**Published:** 1887-07

**Authors:** F. T. Paine

**Affiliations:** Comanche, Texas


### A NOTE ON ELECTRICAL TREATMENT.

*By F. T. Paine, M. D., Comanche, Texas.*

For Daniel's Texas Medical Journal.

YOU will be asked by every applicant for treatment, how long or how many applications will be required to effect a cure. This question you will perhaps never be able to answer satisfactorily. But you can say this to all: Whereas the ordinary sponge electrodes when first applied gave no pain, or were almost entirely inappreciable, you will improve daily in a direct ratio with the increase or improvement in sensation. This is true of all diseased organs, and especially so of the lower half of the person of all females whose menstrual functions are abnormal—more especially the genitalia, including the ovaries and mammary glands. The cure follows the return to electro-sensibility (or according to ERB. *irritability or excitability*) as sure as day and night follow each other. This subject I will elaborate soon.

The wisest plan, which I have been slow to learn, is to refuse all applicants for treatment until a promise is extorted to continue the treatment until the electro-sensibility is fully restored—otherwise no benefit will accrue to either the operator or the patient.

I shall be pleased to correspond with professional gentlemen on all subjects connected with electro-therepeutics.

